# A Flexible and Boron-Doped Carbon Nanotube Film for High-Performance Li Storage

**DOI:** 10.3389/fchem.2019.00832

**Published:** 2019-11-29

**Authors:** Lei Wang, Wenlei Guo, Pengyi Lu, Tao Zhang, Feng Hou, Ji Liang

**Affiliations:** ^1^Key Laboratory of Advanced Ceramics and Machining Technology of the Ministry of Education, School of Materials Science and Engineering, Tianjin University, Tianjin, China; ^2^Institute for Superconducting & Electronic Materials, Australian Institute of Innovative Materials, Innovation, University of Wollongong, North Wollongong, NSW, Australia

**Keywords:** boron-doped, carbon nanotube, flexible, film, lithium storage

## Abstract

Boron-doped carbon nanotubes are a promising candidate for Li storage due to the unique electronic structure and high crystallinity brought by the boron dopants. However, the relatively low Li storage capacity has limited its application in the electrochemical energy storage field, which is mainly caused by the predominantly intact graphitic structure on their surface with limited access points for Li ion entering. Herein, we report a novel B-doped CNTs (py-B-CNTs) film, in which the CNTs possess intrinsically rough surface but flat internal graphitic structure. When used as a flexible anode material for LIBs, this py-B-CNTs film delivers significantly enhanced capacity than the conventional B-doped CNTs or the pristine CNTs films, with good rate capability and excellent cycling performance as well. Moreover, this flexible film also possesses excellent mechanical flexibility, making it capable of being used in a prototype flexible LIB with stable power output upon various bending states.

## Introduction

The demand for high-performance energy storage devices is continuously increasing as a result of the rapid development of portable electronic devices and electric vehicles. Lithium-ion battery (LIB) has been continuously attracting attention in research during the past 30 years and widely applied in a range of applications (Li et al., 2018). However, graphite, the most widely applied commercial anode material for LIBs, is impeding the further improvement of the energy density and power density of LIBs, due to its limited Li storage capacity and hardness of varied structural design (Fang et al., 2017; Liu et al., 2018). Nanostructured carbon materials with highly conductive network provide adequate paths for electron transport; while their abundant pore structures can effectively facilitate the Li ion diffusion and accommodate the volume change of electrodes during an electrochemical process, which is highly beneficial for anode applications for LIBs (Roy and Srivastava, 2015; Tang et al., 2015). Among them, carbon nanotubes (CNTs) are especially promising, because of their superior electrical conductivity, large specific surface area to uniformly load the active substances, and more importantly, the outstanding flexibility for the application for flexible LIBs (Dai, 2002; Kim et al., 2006; Sheem et al., 2006).

Generally, to further enhance the intrinsic Li storage capacity of CNTs, surface modification (Hata et al., 2004; Kang et al., 2012; Lee and Park, 2015), chemical doping (Way and Dahn, 1994; Chen et al., 2013; Sharifi et al., 2015; Zhu et al., 2016), and integration of high capacity active materials (Chen et al., 2008, 2016) are the three major approaches. Among them, doping of heteroatoms [e.g., boron (Stephan et al., 1994; Hsu et al., 2000; Maultzsch et al., 2002; Fujisawa et al., 2018), nitrogen (Maldonado et al., 2006; Bulusheva et al., 2011; Wang L. et al., 2016; Lu et al., 2017), phosphorus (Campos-Delgado et al., 2010; Zhang et al., 2013; Wu et al., 2017) or others] has been widely adopted. On the one hand, the presence of these heteroatoms can introduce more defects, serving as active sites for Li storage with a high capacity. For instance, doping CNTs with nitrogen has been reported to double the Li storage capacity compared with the pristine CNTs (494 vs. 260 mAh g^−1^) (Li et al., 2012). Similar performance enhancement can be achieved by phosphorus or sulfur doping as well (Campos-Delgado et al., 2010; Zhang et al., 2013; Wang C. et al., 2016). On the other hand, heteroatom doping can also provide additional charge carriers that can substantially improve the electronic conductivity of CNTs to achieve a better rate performance for battery applications (Way and Dahn, 1994; Pan et al., 2016).

Among all the doping elements, boron has been considered a quite promising candidate, from several aspects. Firstly, the sizes of boron and carbon atom are similar, which means a minimized lattice distortion to the graphitic structure after doping. This is essential for maintaining the structural stability of the resulted materials during repeated charge-discharge in LIBs (Way and Dahn, 1994; Redlich et al., 1996; Zhang et al., 2016; Geng et al., 2019). Secondly, due to the smaller electronegativity of boron, it will turn the surrounding carbon atom into a negatively-charged center, which attracts more Li ion adsorption (Way and Dahn, 1994). For instance, the boron-doped graphite, which was prepared by annealing pitch coke and boron oxide at 2,800°C, showed a high discharge capacity of 315 mAh g^−1^ at a rate of 1.56 mA cm^−2^ (Tanaka et al., 2001). Thirdly, boron atoms replace the carbon atoms in an sp^2^-type hybrid manner, which supplies more holes to the valence band of carbon, leading to better electronic conductivity of the obtained B-doped carbon materials (Lin et al., 2011; Yeh et al., 2014). Due to this, the boron-doped carbon particles also exhibited better rate capability than the undoped one (Chae et al., 2014). All these unique features of boron doping demonstrate the great potential of boron-doped carbon for Li storage.

Nevertheless, the reports on B-doped CNTs for LIBs are fairly rare, compared with doping them with other elements. One major limitation of the B-doped CNTs is the relatively low capacity, especially at high rates, which is mainly caused by the nearly “intact” graphitic structure with limited access for Li ions to enter the interlayer space as mentioned above. One commonly adopted strategy to tackle with this issue is to create extra surface openings on B-doped carbons, either by mechanical (e.g., shatter or grind) or chemical (e.g., oxidation) methods (Tsang et al., 1994; Eom et al., 2004). Unfortunately, this will inevitably cripple the original framework of B-doped CNTs and compromise their electrochemical performance, especially the cycling stability. In contrast, directly constructing B-doped CNTs with an intrinsically rough surface could be a more attractive method that can not only provide more entrance for Li ions but also maintain the desirable graphitic structure of B-doped CNTs for better electrochemical performance. This is, however, not yet achieved according to the reports by far.

Based on these considerations, we herein report a flexible B-doped CNTs film, which was prepared by a modified floating catalyst chemical vapor deposition (FCCVD) method. By introducing pyridine into the FCCVD method, the morphology of the obtained B-doped CNTs has been successfully altered from a flat surface to a highly rough one, while the inherent graphitic structure remains. As a result, such modified B-doped CNTs (py-B-CNTs) shows much enhanced Li storage performance in comparison with the unmodified B-doped CNTs (B-CNTs), in terms of much-improved capacity, rate capability, and cycling stability. Meanwhile, the as-prepared material also possesses excellent mechanical stability, making it capable of being used in a prototype flexible LIB, which can function well under different bending states. As a result, this flexible and free-standing B-doped CNTs film with excellent Li storage capability has a very good potential to be applied in the next-generation flexible LIBs as well as other battery systems.

## Experimental Section

### Synthesis of py-B-CNTs Film

The py-B-CNTs film was synthesized by a pyridine-modified FCCVD method. Ethanol was chosen as carbon precursor dissolved with ferrocene and thiophene at a mass ratio of 95:1.5:1 (Guo et al., 2019). Then, boric acid (i.e., the boron precursor) and pyridine (i.e., the structure-modifying agent) were added into the precursor solution at proportions of 2 and 4 wt.%, respectively, and ultrasonically agitated for 20 min until fully dissolved. Subsequently, the precursor was treated through an ultrasonic nebulization device and injected into a vertical furnace (1150°C) from the top of the furnace tube. Traveling downwards with the H_2_ carrier gas, the precursor was converted into py-B-CNTs at the hot zone of the furnace and assembled into a film, which was collected continuously at the bottom of the furnace tube. For comparison, non-doped and B-doped CNTs film (denoted CNTs and B-CNTs, respectively) were prepared by unmodified FCCVD method, and non-doped CNTs film (py-CNTs) was prepared through the pyridine-modified FCCVD method as well, without adding boric acid in the precursor.

### Sample Characterization

The tensile strength and resilience of the prepared films were measured by fiber tensile tester (XQ-1C, Shanghai New Fiber Instrument Co., Ltd.) and electronic tensile testing machine (UTM2203, SUNS), respectively. The morphology and microstructure of the films were analyzed by scanning electron microscopy (SEM, S-4800, Hitachi) and transmission electron microscopy (TEM, JEM-2100, JEOL). The chemical state of the materials was studied by X-ray photoelectron spectroscopy (XPS, ESCALAB 250Xi, Thermo Scientific). The infrared spectrum (IR spectrum) was obtained on an infrared spectrometer (Nicolet iS5, ThermoFisher Scientific). The Raman spectra were collected on a Raman spectrometer (LabRAM HR 800, Renishaw) using 532 nm laser. X-ray diffraction (XRD) patterns of NB-CNT films were recorded by a RIGAKU D/Max 2500 Vdiffractometer with Cu Kα radiation source at room temperature. Nitrogen adsorption and desorption isotherms were obtained by at 77 K (ASAP 2020, Micromeritics).

### Electrochemical Measurements

All the samples were cut into an electrode disc (12 mm diameter) by a wafer cutting machine (MSK-T10, HF-Kejing) and used without adding any binders or conductive agents. Coin-type CR2032 cells were assembled in an argon-filled glove box [SUPER (1220/750), Mikrouna, [O_2_] < 0.1 ppm, [H_2_O] < 0.1 ppm], using Li foil as the counter electrode. 1 M LiPF_6_ in ethylene carbonate (EC), diethyl carbonate (DEC) and dimethyl carbonate (DMC) (volume ratio of 1:1:1) solution was used as the electrolyte and a porous film (Celgard, 2400, Celgard) was used as the separator. Galvanostatic charge-discharge measurement was performed on a battery tester (CT-3008W-5V1mA-S4, Neware) over a voltage range of 0.01–3.0 V vs. Li/Li^+^. Cyclic voltammogram (CV) and electrochemical impedance spectroscopy (EIS) tests were carried out on an electrochemical workstation (CHI 660D, CH Instruments). CV was performed over a potential range of 0.01–3.0 V at a scan rate of 0.5 mV s^−1^. EIS was measured in the frequency range of 0.01–10^5^ Hz with a disturbance amplitude of 5 mV. All the experiments were performed at room temperature.

## Results and Discussion

**Figure 1:**
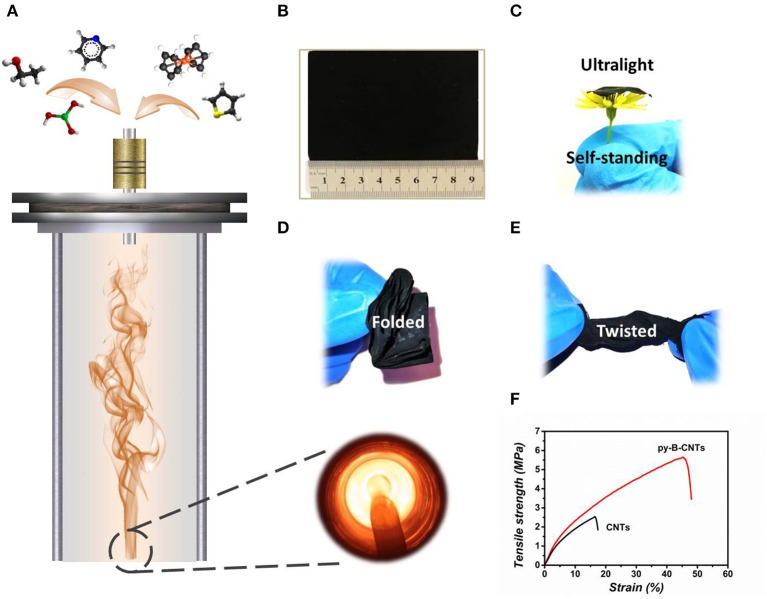
Illustration of the fabrication and flexibility of the py-B-CNTs film. **(A)** Schematic illustration of the synthesis of the py-B-CNTs film from a vertical furnace and corresponding photograph taken from the bottom of the furnace tube; **(B)** optical image of a piece of py-B-CNTs film of ~9 × 7 cm; **(C)** optical image of the ultralight and self-standing film; **(D,E)** optical images of the film under various deformations; and **(F)** tensile strength curves of py-B-CNTs and pristine CNTs films.

The fabrication process of the py-B-CNTs film is illustrated in Figure 1A. The precursor solution, consisting of ethanol, thiophene, ferrocene, boric acid, and pyridine, was gasified through an ultrasonic nebulization device and injected into the vertical furnace tube with an H_2_ atmosphere. The resultant py-B-CNTs film traveled down with the gas flow and was collected at the bottom of the furnace tube. The as-prepared film is light-weight and self-standing, which can withstand various extreme deformation without any structural failures, including being stretched, folded multiple times, and twisted as shown in Figures 1B–E. In particular, the py-B-CNTs film possesses exceptional toughness and stretchability with nearly 50% strain before final fracture (Figure 1F and Figure S1) and high recovery rate of 50% after 10% stretching (Figure S2), which are obviously superior to CNTs film and highly advantageous for flexible LIB applications.

**Figure 2:**
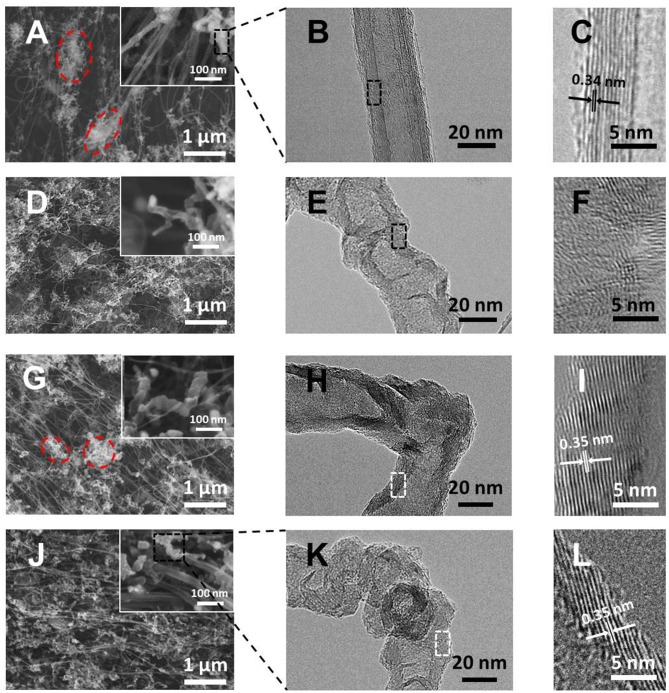
**(A,D,G,J)** SEM images of CNTs, py-CNTs, B-CNTs, and py-B-CNTs films at different magnifications; **(B,E,H,K)** TEM images; and **(C,F,I,L)** high-resolution TEM images of CNTs, py-CNTs, B-CNTs, and py-B-CNTs films, respectively.

The morphology and microstructure of the materials were firstly characterized by SEM and TEM, as shown in Figure 2. On the pristine CNTs film, except for a few clusters, which are typically composed of amorphous carbon (Song et al., 2019) and short crinkly tubes (circled in Figure 2A), the majority of the material are intertwined bunches of long and smooth CNTs (inset of Figure 2A). Under TEM, these long tubes are found to be few-walled CNTs with diameters of 6–8 nm (Figure S3) and the crinkly tubes are multi-walled with a diameter of ~20 nm as well as an average interlayer distance of 0.34 nm, typical of the graphitic structure (Figures 2B,C and Figure S4). In contrast, the involvement of pyridine in the precursor significantly altered the morphology of the obtained CNTs (i.e., py-CNTs). On the one hand, the tangled nanotubes in the pristine CNTs film were scattered into numerous short and curved tubes (Figure 2D), which may be attributed to the reduced interaction forces between short tubes owing to the crinkly surfaces and distorted graphitic structures on their wall as shown in Figures 2E,F and Figure S5 (Nitze et al., 2009). According to previous studies, the appearance of high surface roughness and distorted structures is owing to the distortion to the graphitic structures (e.g., five-member rings in the graphitic framework) introduced by the nitrogen-containing species during the formation of CNTs (Nitze et al., 2009; Yeh et al., 2014). On the other hand, the amount of amorphous carbon around CNTs increased as well. In the case of introducing boron, on the contrary, the obtained B-CNTs film showed another unique morphology, where well-oriented CNTs and rod-like tubes structure were observed (Figure 2G), with an increased diameter up to 30–40 nm in comparison with the pristine CNTs. The graphitic structure of the B-CNTs is, however, seemingly intact with parallel and straight graphitic layers (interlayer distance of 0.35 nm) on its wall (Figures 2H–I), which may be owing to the catalytic graphitization effect of boron on CNTs (Antunes et al., 2006). Specifically, the flat graphitic layers appeared slightly tilted relative to the lumen, thus resulted in a few openings along CNTs and provided extra entrances for Li ions to insert. Moreover, the amorphous carbon wrapped outside nanotubes as observed in both CNTs and py-CNTs almost disappeared, possibly due to the cleaning effect of the water released from the decomposition of boric acid at high temperature (Hata et al., 2004; Cui et al., 2011). However, a few clusters with short tubes were still observable as circled in Figure 2G and Figure S6, probably indicating that boron did not dope in these areas.

In contrast, by modifying the fabrication process using pyridine (i.e., py-B-CNTs), the good alignment of CNTs, which is similar to the B-CNTs, still predominantly remains, with uniformly-distributed crooked tubes surrounding CNTs (Figure 2J). In this case, the py-B-CNTs show a rough surface as in the case of py-CNTs, with the graphitic structure remaining similar to B-CNTs (Figures 2K–L and Figure S7). This clearly demonstrates that the introduction of pyridine in the synthesis of B-CNTs effectively changed the surface morphology of CNTs into a rough one without significantly compromising the graphitic structure of the material. This unique nanostructure of py-B-CNTs with the more exposed surface could provide more access sites for Li ions to enter the tilted graphitic layers of the CNTs to be stored and the intact graphite framework is able to provide enhanced cycling stability in comparison with other defect-rich carbons (Hu et al., 2013; Li et al., 2014; Lin et al., 2014).

**Figure 3:**
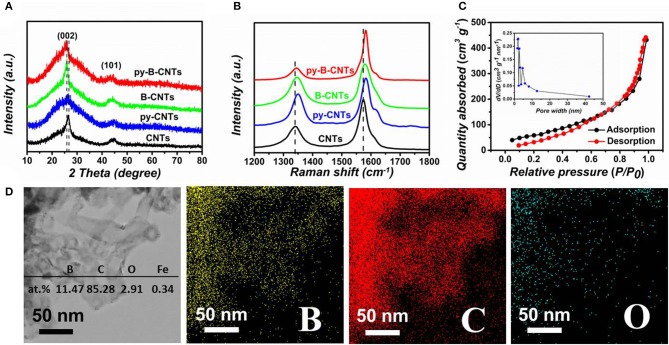
**(A)** XRD patterns and **(B)** Raman spectra of the samples; **(C)** nitrogen adsorption/desorption isotherms with the pore-size distribution (inset) of py-B-CNTs film; **(D)** the EDS elemental analysis of py-B-CNTs and corresponding elemental mapping of the region.

XRD and Raman spectroscopy were then carried out to better study the structure of the material (Figures 3A,B). The typical peaks at around 26°, corresponding to the (002) diffraction, indicate the predominant graphitic structure in CNTs and the latter one at 44° can be attributed to the (101) planes of CNTs. Compared with pristine CNTs, the (002) peak of B-CNTs is narrower and sharper, due to the catalytic graphitization effect of boron, well consistent with the HRTEM observations. Moreover, the slight shift of ~0.9° of (002) peak toward a lower 2θ degree in B-CNTs and py-B-CNTs are observed, indicating a relatively larger interlayer distance than CNTs. By contrast, the broad (002) peak of py-CNTs confirmed the numerous defects created by pyridine additive. At the meantime, the py-B-CNTs also showed a slight shift to lower 2θ degrees but a narrower (002) peak in comparison with that of py-CNTs, due to the B doping and pyridine addition, comprehensively.

More structural information of the materials was obtained from Raman spectra (Figure 3B). The D band at 1,346 cm^−1^ is associated with defects and distortions in the graphitic framework; while the G band at 1,579 cm^−1^ corresponds to the graphitic layers and stretching vibration of carbon atoms (Yang et al., 2017). A slight shift to the higher wavenumber of the G-band for B-CNTs, py-CNTs, and py-B-CNTs is observed in comparison with pristine CNTs, which might be due to the modification in the electronic structure of CNTs (Handuja et al., 2009; Koós et al., 2010). On the other hand, the up-shift of D-band indicates the appearance of new types of disorders or chemical bonds (i.e., B-C or B-O bonds) in B-CNTs, py-CNTs, and py-B-CNTs compared with those of pristine CNTs (Bulusheva et al., 2011), while the shoulder at 1616 cm^−1^ (i.e., D' band) for py-CNTs should be ascribed to the slightly damaged graphite structure (Antunes et al., 2006). In addition, the intensity ratios of the D and G bands (I_D_/I_G_) of py-B-CNTs, B-CNTs, CNTs, and py-CNTs were calculated to be 0.43, 0.89, 0.95, and 1.12, respectively. The relatively lower I_D_/I_G_ ratio of B-CNTs can be attributed to the removal of amorphous carbon and catalytic graphitization effect of B on CNTs, while the higher I_D_/I_G_ of py-CNTs is mainly owing to the defects created by pyridine and increased amorphous carbon compared with pristine CNTs. Remarkably, py-B-CNTs showed the smallest I_D_/I_G_, possibly owing to the loosening of the tangled tubes as in B-CNTs and the more thorough catalytic graphitization from boron.

To further investigate the pore structure of the materials, nitrogen adsorption and desorption isotherms were obtained (Figure 3C and Figure S8). The specific surface area of py-B-CNTs was calculated to be 229.8 m^2^ g^−1^, and a combined type III and type IV isotherm with hysteresis loop at medium to high pressure regions was obtained. This indicates the existence of small amounts of mesopores or macropores in py-B-CNTs, which may originate from the arrangement of the intertwined nanotubes (Ding et al., 2017). The pore size distribution in the inset of Figure 3C further showed that these small mesopores are mainly 1.6–2.4 nm, which may originate from the interspaces between tangled nanotubes and long CNTs in py-B-CNTs. Similar results can also be observed in py-CNTs, B-CNTs, and pristine CNTs, as shown in Figure S8. The corresponding specific surface areas were calculated to be 198.8, 137.1, and 127.2 m^2^ g^−1^, respectively. Based on this, the high surface area of py-B-CNTs is mainly owing to the rough surface as well as scattered clusters induced by pyridine and the removal of amorphous carbon during the fabrication. The large surface area of py-B-CNTs would provide abundant access sites for Li ions during the charge/discharge process in LIBs.

The materials' chemical composition was then studied by energy dispersive spectroscopy (Figure 3D and Table S1), and it was measured that content of B, C, O, Fe was 11.47, 85.28, 2.91, and 0.34 at.%, respectively, for py-B-CNTs film and no nitrogen was detected. For other materials, py-CNTs possessed higher O content of 5.66 at.% than B-CNTs (0.90 at.%) and CNTs (1.16 at.%), attributed to the easier oxygen adsorption on the defect sites (Yang et al., 2011; Song et al., 2019). The elemental mapping of the materials (Figure 3D and Figures S9, S10) reveals the uniform distribution of carbon, boron, and oxygen over the whole material. The absence of nitrogen indicates that pyridine only participated in the CNTs formation process and changed the morphology of CNTs, but did not cause N-doping in the CNTs, which is also in agreement with the EDS results and has been observed in our previous studies (Song et al., 2019). The chemical states of the elements in py-B-CNTs and other materials were further characterized by XPS survey spectrums (Figures S11–S14). Especially, in the high-resolution spectrums, the total amount of oxygen-containing functional groups (C-OH, C = O, COOH) on py-B-CNTs is obvious higher than on B-CNTs, demonstrating the active sites effectively introduced by pyridine, which is advantageous for Li storage. Similar results can be observed for py-CNTs and CNTs in high-resolution XPS (Figures S13, S14).

**Figure 4:**
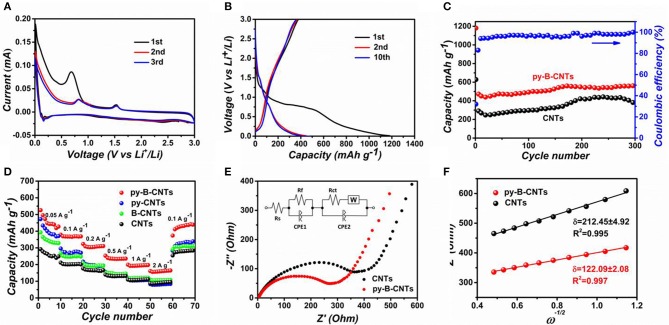
**(A)** CV loops of the py-B-CNTs film obtained at 0.1 mV s^−1^; **(B)** galvanostatic charge-discharge (GCD) profiles of py-B-CNTs film; **(C)** cycling performances of the py-B-CNTs and pristine CNTs films for 300 cycles at 100 mA g^−1^; **(D)** rate capability of the materials at various rates of 0.05-2 A g^−1^; **(E)** electrochemical impedance spectra of py-B-CNTs and pristine CNTs films and the equivalent circuit to fit the EIS; **(F)** the fitted curve of impedance (Z′) vs. the reciprocal square root of the angular frequency (ω) for py-B-CNTs film.

To characterize the electrochemical performances of py-B-CNTs film and the analog materials, they were assembled in a coin cell using a Li foil as the counter electrode. CV loops of py-B-CNTs and CNTs film of the first three scanning cycles are presented in Figure 4A and Figure S15. For both materials, in the first discharge process of the materials, a strong peak appeared at 0.7 V, which disappeared in the subsequent cycles. This is caused by the decomposition of the electrolyte to form the solid electrolyte interface (SEI) that prevents further contact between electrodes and electrolyte, causing an irreversible capacity. On the other hand, the peak at around 0.2 V is related to the Li ion intercalation process into the graphitic structure of the materials (Chae et al., 2014). Compared with the pristine CNTs, two extra peaks at 0.8 and 1.5 V are observed for py-B-CNTs. The former one, which was also observed in py-CNTs and B-CNTs (Figure S16), might be attributed to the defects created by pyridine additive during the fabrication of CNTs to serve as Li storage sites (Pan et al., 2016), or the existence of new type of chemical bond (e.g., B-O bond) (Chae et al., 2014). As for the other peak at 1.5 V, which was also seen in B-CNTs (Figure S16), it should be attributed to the formation of Li_x_(B_z_C_l−z_)_6_ species, allowing more Li ions to be stored (Way and Dahn, 1994; Chae et al., 2014). As previously discussed, boron acts as an electron acceptor in the carbon lattice and Li will donate its 2s electrons when intercalated into the carbon host (Redlich et al., 1996). Therefore, the introduction of boron strengthens the chemical bond between the intercalated Li ion and the B-doped carbon host, compared to the pure carbon host (Way and Dahn, 1994). In the subsequent cycles, the curves were almost overlapped, demonstrating the good electrochemical stability of py-B-CNTs film anodes.

The GCD profiles of py-B-CNTs film anode in the first, second, and tenth cycles at the current density of 100 mA g^−1^ are shown in Figure 4B. In the first discharge process, the potential plateau at ~0.8 V is ascribed to the formation of the SEI, which is consistent with the CV results. The initial discharge and charge capacities are 1182 and 377 mAh g^−1^, corresponding to an initial Coulombic efficiency of 32%. The large irreversible capacity is also reflected on py-CNTs, B-CNTs, and CNTs anodes (initial Coulombic efficiency of 35, 39, and 43%, respectively) as shown in Figure S17, which mainly result from the decomposition of the electrolyte during the formation of SEI on the large surface of the CNTs films (Zhang et al., 2016; Lu et al., 2017). In the following cycles, the Coulombic efficiency increased to 83% in the second cycle and over 97% in the tenth cycle, indicating the high reversibility of py-B-CNTs film electrode.

In order to further measure the cycling performance of py-B-CNTs and CNTs films, the anodes were tested at 100 mA g^−1^ for 300 cycles. As shown in Figure 4C, the py-B-CNTs film delivered a high reversible capacity of 548 mAh g^−1^ after 300 cycles, which is significantly higher than that of CNTs, and the columbic efficiency was always over 97%. The excellent cycling performance of py-B-CNTs film mainly benefitted from the high conductivity and stably doped B atoms in the py-B-CNTs. It is noticeable that the capacities of both electrodes slightly increased along with the number of cycles, which could be attributed to two main reasons. On the one hand, the nature of porous films results in the gradual infiltration of electrolyte for active materials. On the other hand, the interlayer spacing of CNTs becomes more expanded with the continuous insertion-extraction of Li ions, which is advantageous to the further Li ion transport between electrodes and the electrolyte and has been also observed in other previous reports (Li et al., 2012; Wang L. et al., 2016; Lu et al., 2017). Besides, a downward trend of the CNTs anode appeared after 250 cycles due to the irreversible lithiation-delithiation process caused by the channel-block phenomenon (Pan et al., 2016), which does not exist for py-B-CNTs anode because of the more abundant entrance sites on the py-B-CNTs, as previously discussed (Wang L. et al., 2016).

The rate capacities of the materials were evaluated at various current densities and compared in Figure 4D. Among these materials, the py-B-CNTs film delivered specific capacities of 474, 378, 312, 235, 198, and 167 mAh g^−1^ at current densities from 0.05 to 2 A g^−1^, which were much higher than those of py-CNTs, B-CNTs, and CNTs films. The relatively lower capacities of B-CNTs than py-B-CNTs might be owing to that the graphitic layers was not active for Li ions adsorption while introducing extra defects at the openings could ameliorate this. Moreover, it is noted that py-B-CNTs also showed higher capacity retention than py-CNTs at high current densities (56% for py-B-CNTs and 39% for py-CNTs from 0.2 to 2 A g^−1^), which is due to the improved electrical conductivity by boron doping. Similar results can also be found in the B-CNTs and CNTs samples. Moreover, all the electrodes showed good recovery capability when the rate was switched back to 0.1 A g^−1^, benefiting from the stability of prepared films.

To further confirm the advantages in the kinetics of the electrochemical process of the py-B-CNTs film, EIS measurement was carried out on the py-B-CNTs and CNTs (Figure 4E). It is observed that the Nyquist plots of both materials consist of a depressed semicircle in the high-frequency region and a linear Warburg part in the low frequency region, attributed to charge-transfer resistance and Li-ion diffusion in CNTs network structure, respectively (Sang et al., 2018). Specifically, an equivalent circuit was fitted as shown in the inset of Figure 4E. R_s_ denotes the ohmic resistance of electrolyte, while R_f_ and R_ct_ correspond to SEI film resistance and charge-transfer resistance. CPE1 and CPE2 represent SEI film capacitance and double layer capacitance, while W is the finite length Warburg impedance related to the solid-state diffusion in the electrodes. The fitted R_ct_ value of py-B-CNTs is 264 Ω, lower than CNTs (373 Ω), indicating a lower charge-transfer resistance in the py-B-CNTs film anode, benefiting from the uniform and well-oriented microstructure of py-B-CNTs film. Besides, the corresponding charge-transfer resistance of B-CNTs and py-CNTs films can also be fitted as 109 and 664 Ω, respectively (Figure S18). Moreover, the influence factors for Li-ion diffusion efficiency (D_Li_) was calculated by Equation 1, in which R is the gas constant, T is the absolute temperature, A is the surface area of the electrode, n is the number of electrons per molecule during oxidization, F is the Faraday constant, C is the concentration of the Li ion, and δ is the Warburg factor (Jin et al., 2017):

(1) $$\begin{array}{l} D_{Li}\;=\;\frac{R^{2}T^{2}}{2A^{2}n^{4}F^{4}C^{2}\delta^{2}} \end{array}$$

Among these, δ can be obtained according to the slope of the fitting curve referring to the real impedance (Z′) and angular frequency (ω) as shown in Figure 4F. Obviously, the smaller δ value for py-B-CNTs film anode reflects faster Li-ion diffusion process compared with CNTs film anode.

**Figure 5:**
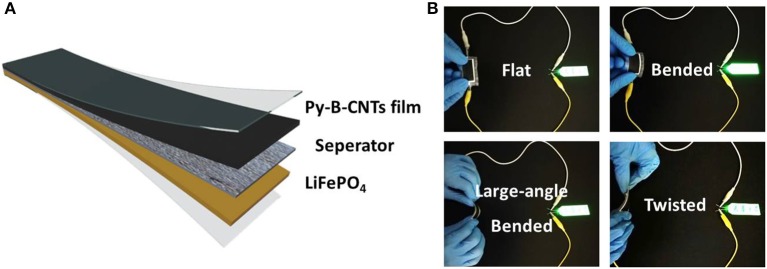
**(A)** Schematic illustration of the flexible LIB with py-B-CNTs film and commercial LiFePO_4_ as electrodes; **(B)** photographs of the flexible LIB powering a light-emitting diode board under various bending conditions.

To further demonstrate the feasibility of py-B-CNTs film for flexible electronic devices, flexible LIBs was assembled with commercial LiFePO_4_ cathode as illustrated in Figure 5A. As shown in Figure 5B, after being charged to 3.5 V, the flexible LIB could stably work to power a light-emitting diode (LED) board, regardless of a series of large-angle bending and twisting, demonstrating the superior conductivity and mechanical stability by boron doping, effectively promising its applications in flexible electronics.

Based on the measurements and analysis above, the unique microstructure and excellent electrochemical performance of py-B-CNTs film can be attributed to several reasons. Firstly, the doping of boron in the carbon framework supplied more holes to the valence band and led to the better electronic conductivity of CNTs. Secondly, the catalytic graphitization of B guaranteed the cycling stability of CNTs during rapid intercalation/deintercalation of Li ion process. Moreover, the surface modification created by pyridine provides more access sites for Li ions to enter the graphitic layers along the CNTs. These factors should comprehensively endow the py-B-CNTs film anodes with outstanding rate capability and cycling stability, as well as excellent toughness for flexible LIBs application.

## Conclusions

In summary, a highly flexible and free-standing py-B-CNTs film was facilely prepared via one-step FCCVD method. The introduction of B endowed CNTs with large surface area as well as high graphitization degree and the involvement of pyridine in the growth of CNTs created abundant active sites for Li storage. The unique structure of py-B-CNTs film is conducive to the high specific capacity with excellent cycle performance of 548 mAh g^−1^ after 300 cycles at 0.1 A g^−1^. Moreover, the py-B-CNTs film could endure nearly 50% strain without any fractures, which guaranteed its desirable application in full flexible LIBs.

## Data Availability Statement

All datasets generated for this study are included in the article/Supplementary Material.

## Author Contributions

LW designed this project and was in charge of the analysis of data and drafting of the work. WG, PL, and TZ prepared samples and performed tensile test. All the authors contributed to the manuscript preparation.

### Conflict of Interest

The authors declare that the research was conducted in the absence of any commercial or financial relationships that could be construed as a potential conflict of interest.
