# Supplementary material for: A Flexible and Boron-Doped Carbon Nanotube Film for High-Performance Li Storage
Source: Front Chem. 2019 Nov 29;7:832. doi: 10.3389/fchem.2019.00832 (PMC6897285; doi:10.3389/fchem.2019.00832)
Supplement: Supplementary file 1 [file Table_1.DOCX]

Supplementary Material

**A Flexible and Boron-doped Carbon Nanotube Film for High-performance Li Storage**

**Lei Wang ^a^, Wenlei Guo ^a^, Pengyi Lu ^a^, Tao Zhang ^a^, Feng Hou ^a^ *, and Ji Liang ^a, b^ ****

^a^ Key Laboratory of Advanced Ceramics and Machining Technology of the Ministry of Education, School of Materials Science and Engineering, Tianjin University, Tianjin 300072, China.

^b^ Institute for Superconducting & Electronic Materials, Australian Institute of Innovative Materials, University of Wollongong, Innovation Campus, Squires Way, North Wollongong, NSW 2500, Australia

* **Correspondence:**

* Corresponding Authors.

** Corresponding Authors

*E-mail Addresses:* houf@tju.edu.cn (F. Hou); liangj@uow.edu.au (J. Liang)

# Supplementary Figures and Tables

**
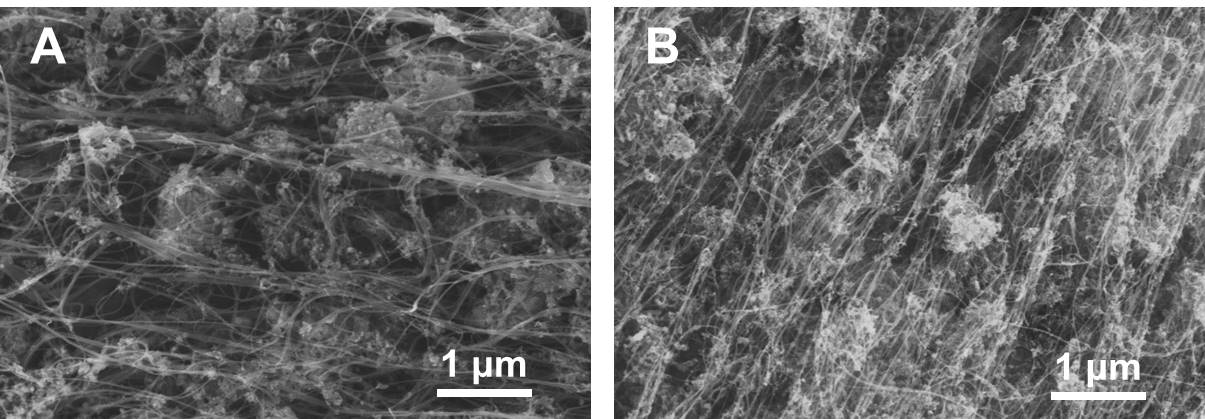
**

**Figure. S1.** The microstructure characterizations of py-B-CNTs film **(A)** before and **(B)** after stretching test.


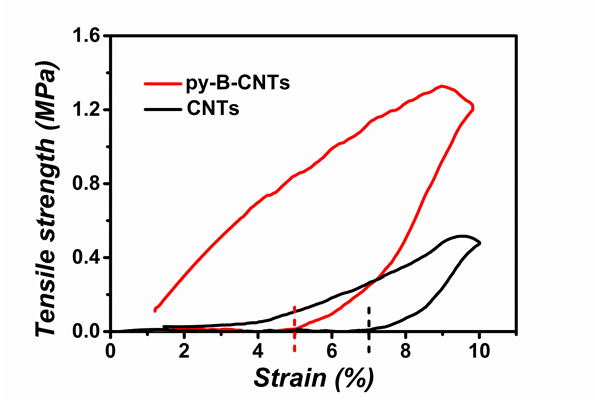


**Figure S2.** The recovery properties of py-B-CNTs and CNTs films after stretching tests.


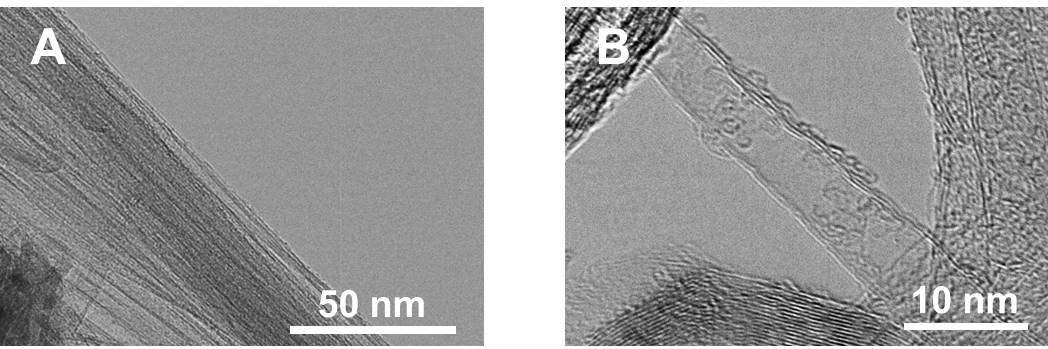


**Figure S3.** The TEM images of long tubes in CNTs film.


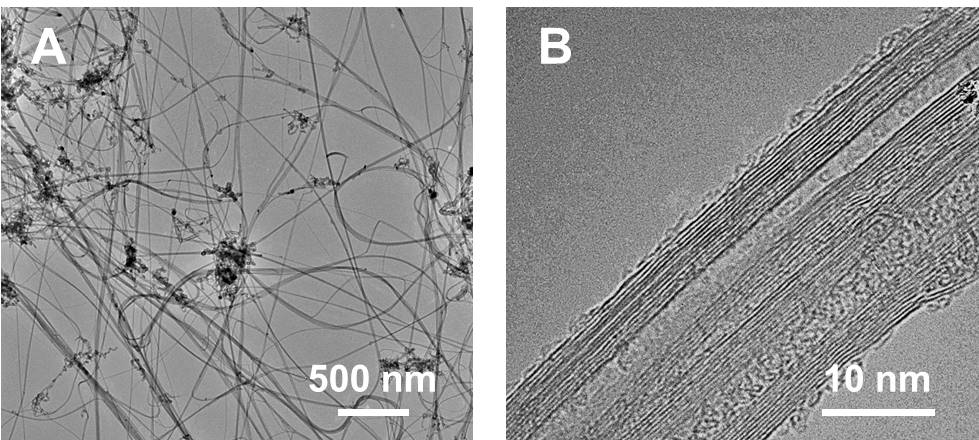


**Figure S4.** The TEM images of CNTs film at **(A)** low and **(B)** high magnification.


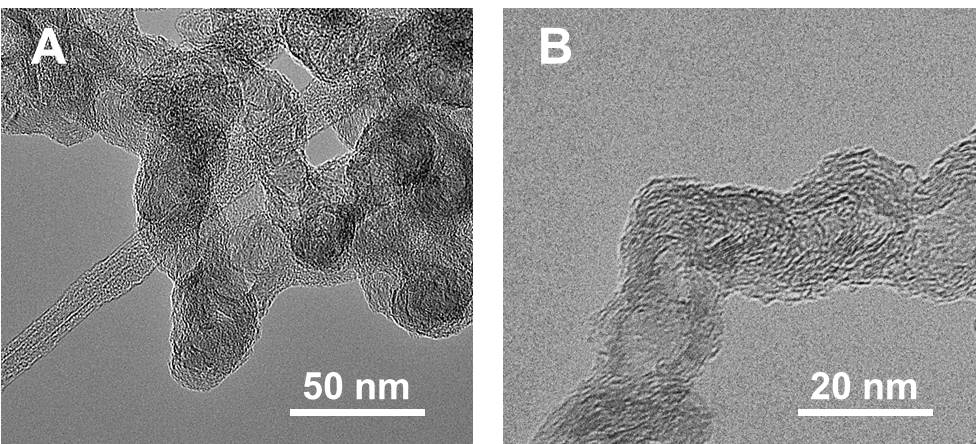


**Figure S5.** The TEM images of py-CNTs film.


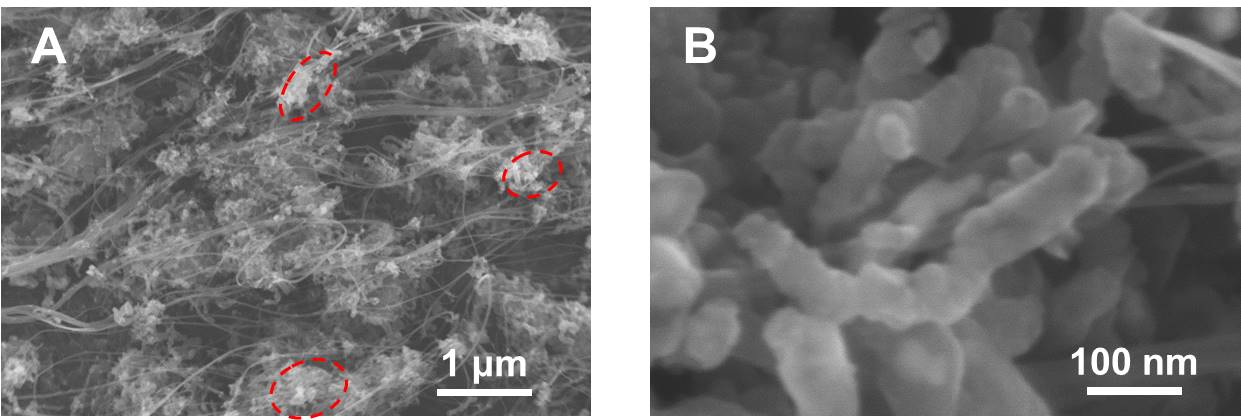


**Figure S6.** The SEM images of B-CNTs film.


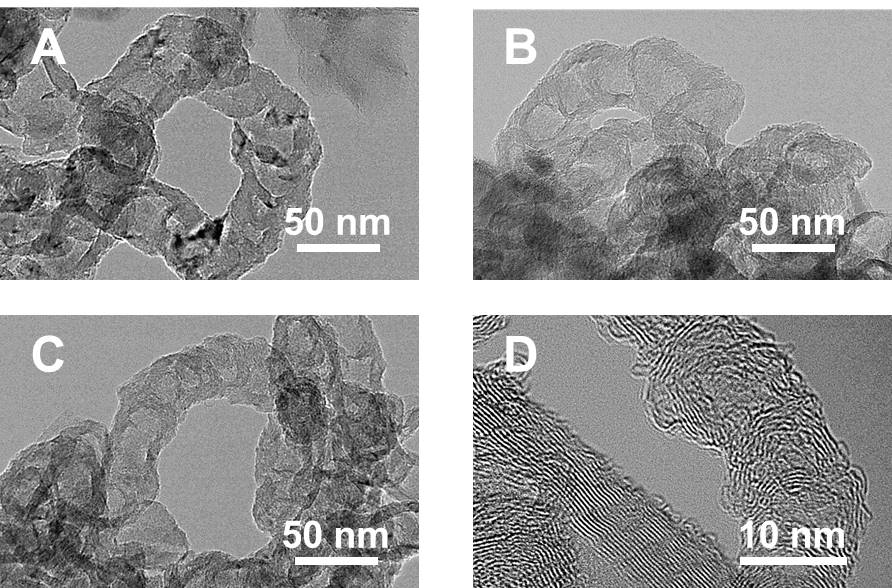


**Figure S7.** The TEM images of py-B-CNTs film.


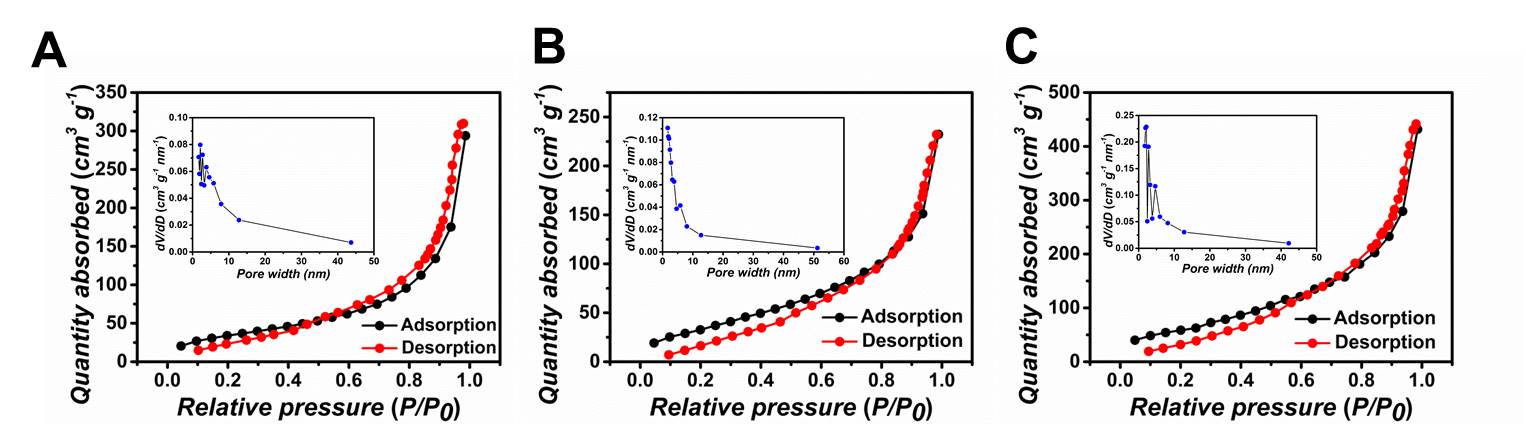


**Figure S8.** N2 adsorption-desorption isotherm and the corresponding pore size distribution of **(A)** CNTs, **(B)** B-CNTs and **(C)** py-CNTs films.

**Table S1.** Elemental analysis of the prepared films.

**
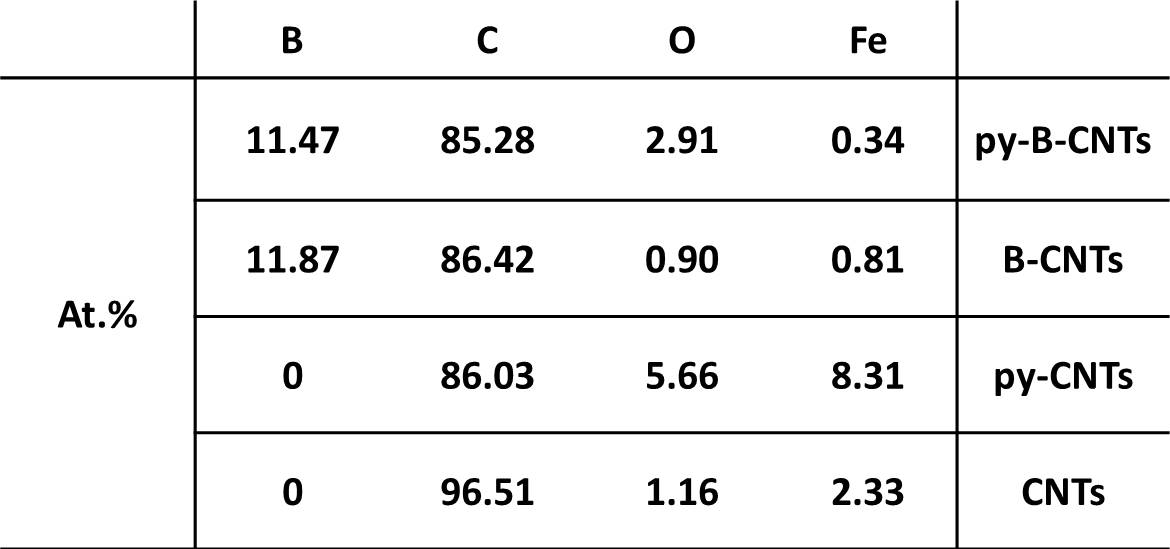
**


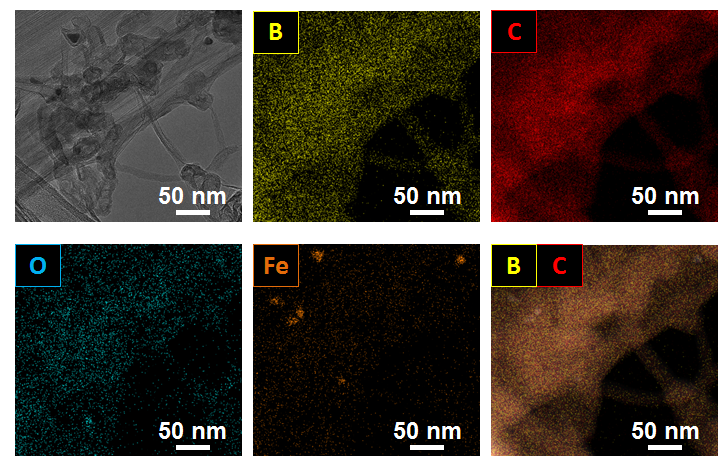


**Figure S9.** The corresponding elemental mapping of various elements in B-CNTs film.


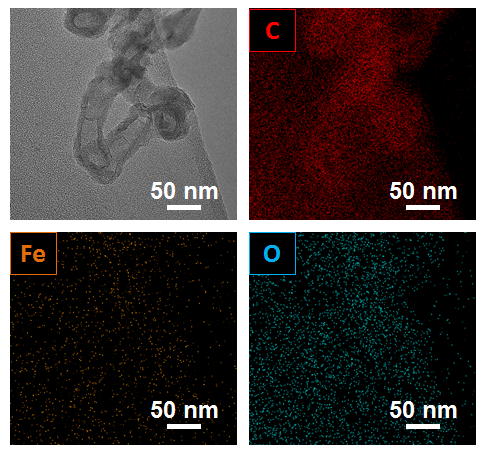


**Figure S10.** The corresponding elemental mapping of various elements in py-CNTs film.

**
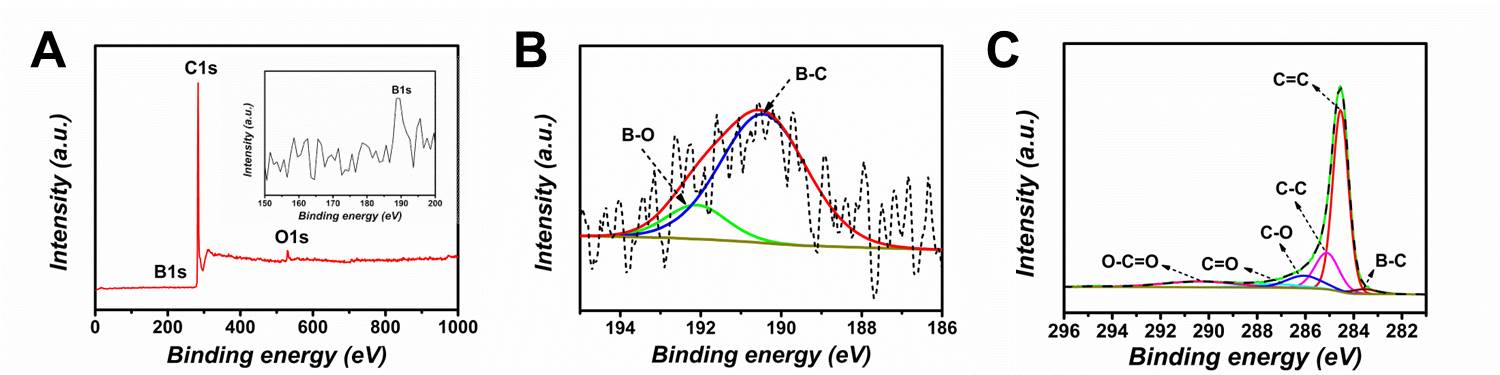
**

**Figure S11. (A)** The survey scan XPS spectra and **(B)** high resolution B1s, **(C)** C1s spectrum of py-B-CNTs films.

**
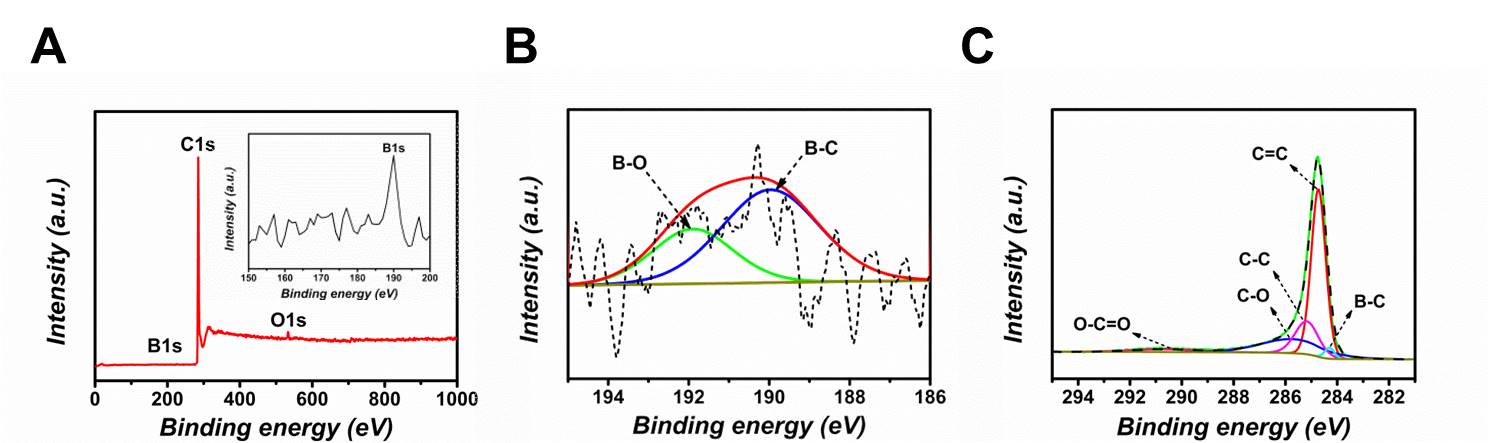
**

**Figure S12. (A)** The survey scan XPS spectra and **(B)** high resolution B1s, **(C)** C1s spectrum of B-CNTs films.

**
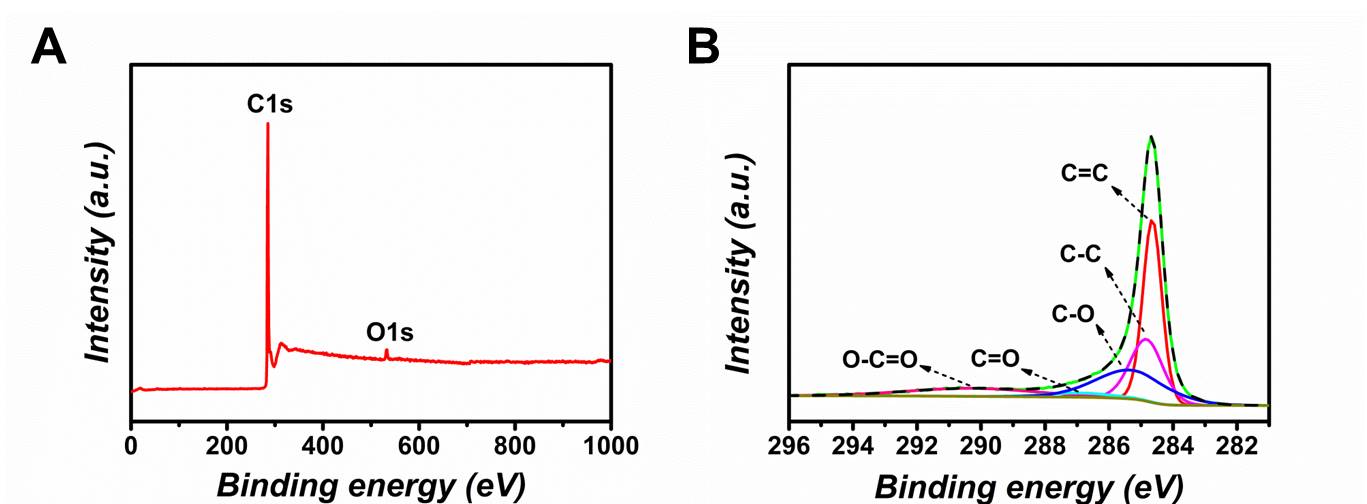
**

**Figure S13. (A)** The survey scan XPS spectra and **(B)** high resolution C1s spectrum of py-CNTs films.

**
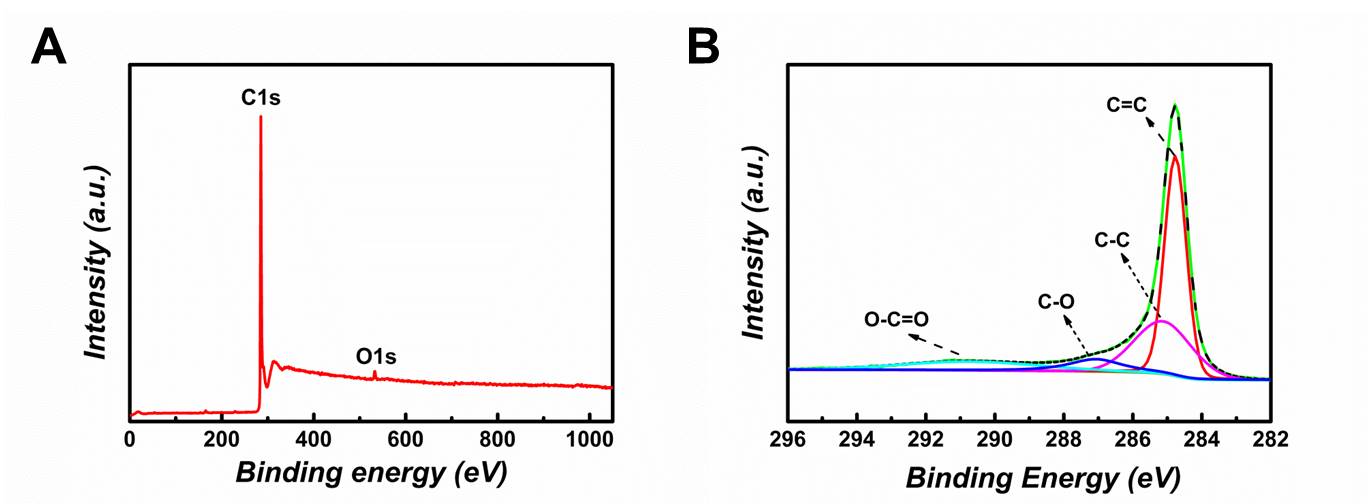
**

**Figure S14. (A)** The survey scan XPS spectra and **(B)** high resolution C1s spectrum of CNTs films.

**
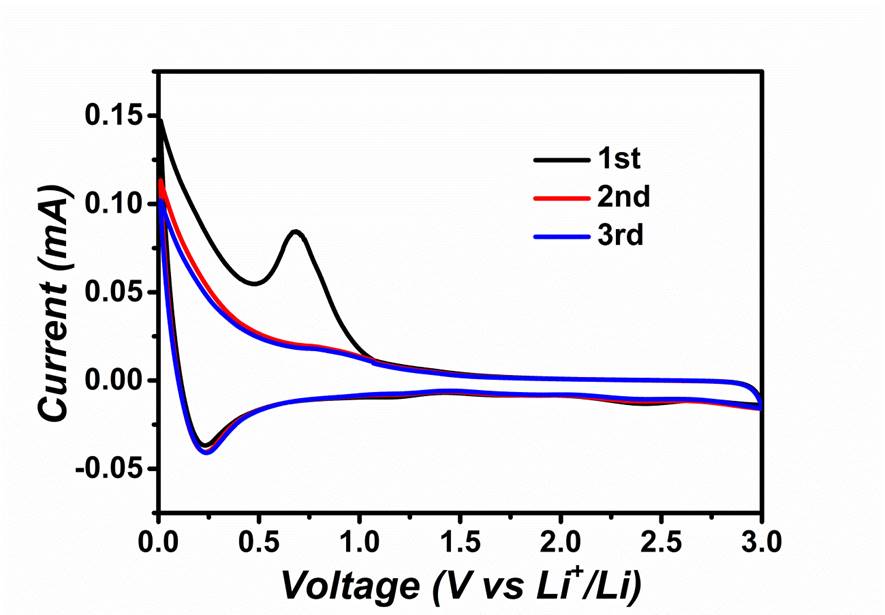
**

**Figure S15.** The CV curve of CNTs film at a scan rate of 0.1 mV s^-1^.

**
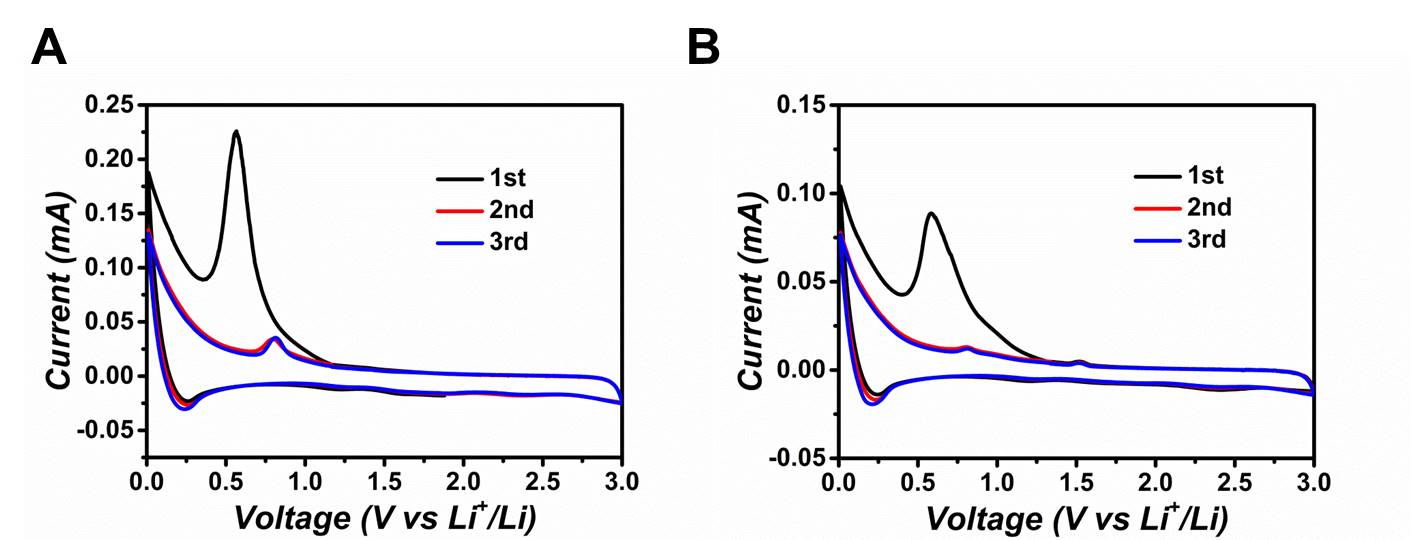
**

**Figure S16.** The CV curves of **(A)** py-CNTs and **(B)** B-CNTs films at a scan rate of 0.1 mV s^-1^.

**
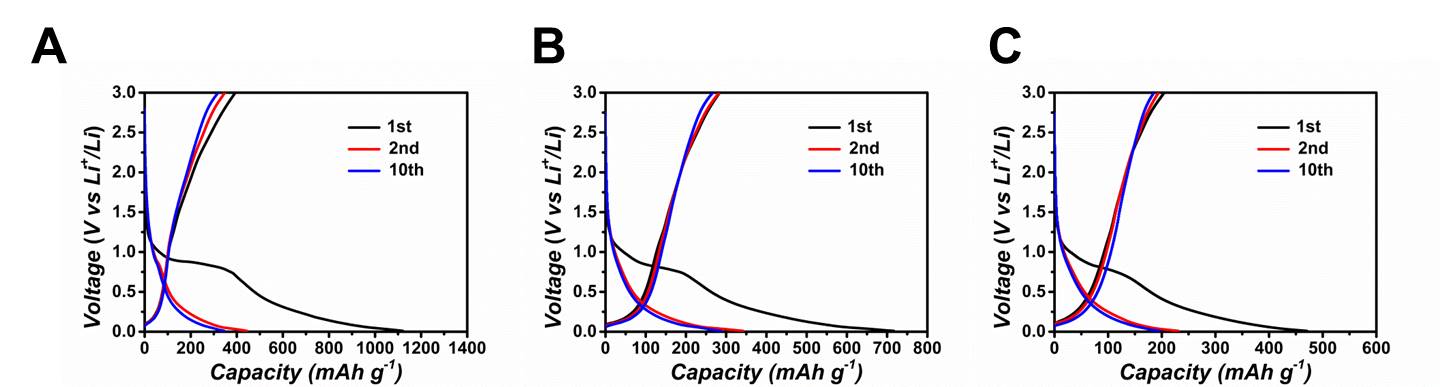
**

**Figure S17.** The GCD curve of **(A)** py-CNTs, **(B)** B-CNTs and **(C)** CNTs film at the current density of 100 mA g^-1^.

**
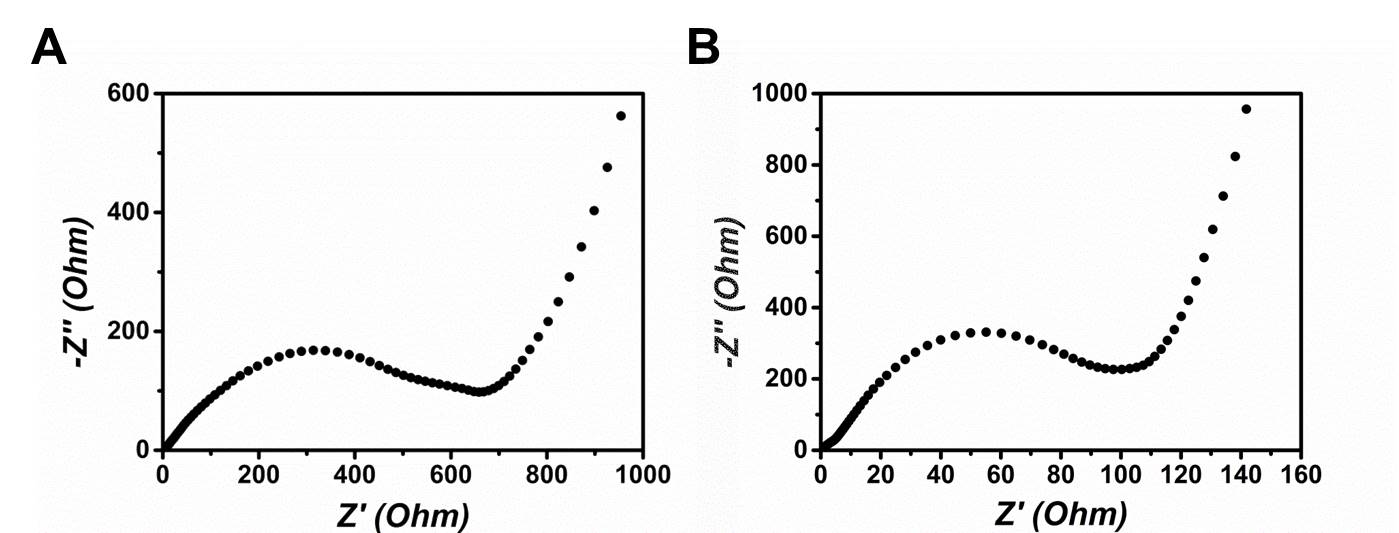
**

**Figure S18. a)** The Nyquist plots of **(A)** py-CNTs and **(B)** B-CNTs films anodes.
